# Subversion of Metabolic Wasting as the Mechanism for *folM*-Linked Sulfamethoxazole Resistance

**DOI:** 10.1128/mBio.01769-17

**Published:** 2017-11-28

**Authors:** Yusuke Minato, Anthony D. Baughn

**Affiliations:** Department of Microbiology and Immunology, University of Minnesota Medical School, Minneapolis, Minnesota, USA; University of British Columbia

**Keywords:** *Burkholderia*, antifolate drugs, antimicrobial agents, Bactrim, co-trimoxazole, drug resistance, folate metabolism, sulfamethoxazole, trimethoprim

## LETTER

In their recent paper (1), Podnecky et al. identified novel clinically relevant co-trimoxazole resistance mutations in *Burkholderia pseudomallei*, the causative agent of melioidosis. Co-trimoxazole, a combination of sulfamethoxazole (SMX) and trimethoprim (TMP), is the best-studied and most widely used synergistic antimicrobial drug combination and is an essential component of melioidosis treatment. Podnecky et al. identified mutations in *bpeT* and *bpeS* from laboratory and clinical co-trimoxazole-resistant isolates of *B. pseudomallei*. Their elegant work demonstrated that mutations in *bpeT* or *bpeS* result in constitutive expression of the BpeEF-OprC efflux pump that confers co-trimoxazole resistance. The authors also provide the first report of *folM* mutations that confer SMX monoresistance, and yet, the biochemical basis for this novel molecular resistance mechanism was not fully explained.

**FIG 1:**
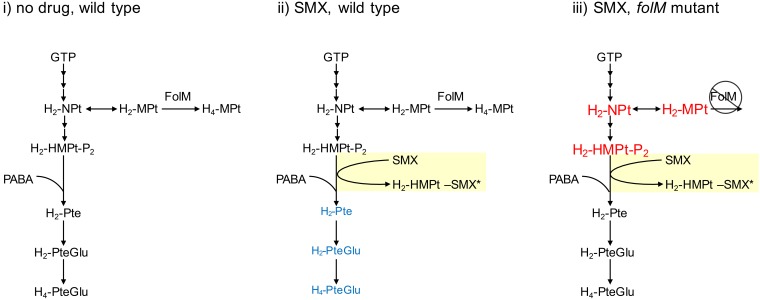
Branched pathway for tetrahydrofolate and tetrahydromonapterin synthesis in *B. pseudomallei* (modified from Fig. 1 in the work of Podnecky et al. [1]). Blue text in scheme ii represents native metabolites that are expected to decrease in abundance relative to scheme i following treatment with SMX. Red text in scheme iii represents native metabolites that are expected to increase in abundance relative to scheme i due to *folM* mutation. The asterisk indicates an unmetabolizable product of SMX metabolism. Abbreviations: H_2_-NPt, 7,8-dihydroneopterin; H_2_-MPt, 7,8-dihydromonapterin; H_4_-MPt, tetrahydromonapterin; H_2_-HMPt-P_2_, 6-hydroxymethyl-7,8-dihydropterin pyrophosphate; PABA, *para*-aminobenzoic acid; H_2_-Pte, dihydropteroate; H_2_-PteGlu, dihydrofolate; H_4_-PteGlu, tetrahydrofolate; SMX, sulfamethoxazole.

*folM* encodes dihydromonapterin reductase that catalyzes the final step in synthesis of tetrahydromonapterin (H_4_-MPt), a nonessential branched pathway from the folate biosynthesis pathway (Fig. 1). H_4_-MPt is a major pterin produced by *Escherichia coli* and likely many other bacterial species (2). Loss-of-function mutations in *folM* are expected to result in increased metabolic flux toward synthesis of the folate precursor dihydropterin pyrophosphate (H_2_-HMPt-P_2_).

SMX is typically regarded as an inhibitor of dihydropteroate synthase (FolP). However, it was recently demonstrated that SMX acts instead by competing with *para*-aminobenzoic acid (PABA) for ligation with H_2_-HMPt-P_2_ (Fig. 1) (3, 4). As a result, SMX forms dead-end complexes with H_2_-HMPt-P_2_ (H_2_-HMPt-SMX) and depletes the H_2_-HMPt-P_2_ pool and thereby inhibits dihydropteroate production through metabolic wasting (3–5). Consequently, SMX susceptibility is not impacted by the amount of “target” enzyme but is primarily influenced by the intracellular abundance of its cosubstrates PABA and H_2_-HMPt-P_2_. In contrast, the activity of TMP, a competitive inhibitor of dihydrofolate reductase, can be affected by both the intracellular abundance of substrate (dihydrofolate) and the amount of target enzyme.

We propose that the loss-of-function mutations in *folM* confer SMX resistance by increasing H_2_-HMPt-P_2_ production that mitigates SMX-driven metabolic wasting (Fig. 1). H_2_-HMPt-P_2_ overproduction is not sufficient to confer resistance to TMP because an equivalent amount of PABA would be required to increase dihydrofolate production. Based on this understanding of factors that govern susceptibility and resistance to SMX and TMP, we think that it is important to determine whether H_2_-HMPt-P_2_ is overproduced in the *folM* mutant. Further, it would seem worthwhile to determine whether *folM* mutations can confer resistance to TMP in PABA-overproducing strains. Resolving these standing questions is likely to reveal the biochemical basis for this novel antifolate drug resistance mechanism.

## References

[1] Podnecky NL, Rhodes KA, Mima T, Drew HR, Chirakul S, Wuthiekanun V, Schupp JM, Sarovich DS, Currie BJ, Keim P, Schweizer HP 2017 Mechanisms of resistance to folate pathway inhibitors in *Burkholderia pseudomallei*: deviation from the norm. mBio 8:e01357-17. doi:10.1128/mBio.01357-17. 28874476 PMC5587915

[2] Pribat A, Blaby IK, Lara-Núñez A, Gregory JF, de Crécy-Lagard V, Hanson AD 2010 FolX and FolM are essential for tetrahydromonapterin synthesis in Escherichia coli and Pseudomonas aeruginosa. J Bacteriol 192:475–482. doi:10.1128/JB.01198-09. 19897652 PMC2805310

[3] Palmer AC, Kishony R 2014 Opposing effects of target overexpression reveal drug mechanisms. Nat Commun 5:4296. doi:10.1038/ncomms5296. 24980690 PMC4408919

[4] Yun MK, Wu Y, Li Z, Zhao Y, Waddell MB, Ferreira AM, Lee RE, Bashford D, White SW 2012 Catalysis and sulfa drug resistance in dihydropteroate synthase. Science 335:1110–1114. doi:10.1126/science.1214641. 22383850 PMC3531234

[5] Bock L, Miller GH, Schaper KJ, Seydel JK 1974 Sulfonamide structure-activity relationships in a cell-free system. 2. Proof for the formation of a sulfonamide-containing folate analog. J Med Chem 17:23–28. doi:10.1021/jm00247a006. 4357096

